# Providing Students with Adequate School Drinking Water Access in an Era of Aging Infrastructure: A Mixed Methods Investigation

**DOI:** 10.3390/ijerph17010062

**Published:** 2019-12-20

**Authors:** Erica L. Kenney, James G. Daly, Rebekka M. Lee, Rebecca S. Mozaffarian, Katherine Walsh, Jill Carter, Steven L. Gortmaker

**Affiliations:** 1 Department of Nutrition, Harvard T.H. Chan School of Public Health, 665 Huntington Avenue, Boston, MA 02115, USA; 2 Department of Social and Behavioral Sciences, Harvard T.H. Chan School of Public Health, 677 Huntington Avenue, Boston, MA 02115, USA; jdaly@hsph.harvard.edu (J.G.D.); rlee@hsph.harvard.edu (R.M.L.); rmozaffa@hsph.harvard.edu (R.S.M.); sgortmak@hsph.harvard.edu (S.L.G.); 3 Department of Facilities Management, Boston Public Schools, 1216 Dorchester Ave, Dorchester, MA 02125, USA; kwalsh4@bostonpublicschools.org; 4 Office of Social Emotional Learning and Wellness Instruction & Policy, Boston Public Schools, 2300 Washington Street, Roxbury, MA 02119, USA; jcarter@bostonpublicschools.org

**Keywords:** schools, drinking water, infrastructure, water safety, water consumption

## Abstract

Ensuring students’ access to safe drinking water at school is essential. However, many schools struggle with aging infrastructure and subsequent water safety problems and have turned to bottled water delivery systems. Little is known about whether such systems are feasible and effective in providing adequate student water access. This study was a mixed-methods investigation among six schools in an urban district in the U.S. with two types of water delivery systems: (1) tap water infrastructure, with updated water fountains and bottle fillers, and (2) bottled water coolers. We measured students’ water consumption and collected qualitative data from students and teachers about their perceptions of school drinking water. Student water consumption was low—between 2.0 (SD: 1.4) ounces per student and 2.4 (SD: 1.1) ounces per student during lunch. Students and teachers reported substantial operational hurdles for relying on bottled water as a school’s primary source of drinking water, including difficulties in stocking, cleaning, and maintaining the units. While students and teachers perceived newer bottle filler units positively, they also reported a distrust of tap water. Bottled water delivery systems may not be effective long-term solutions for providing adequate school drinking water access and robust efforts are needed to restore trust in tap water.

## 1. Introduction

Promoting the consumption of plain water as a beverage and promoting better access to drinking water are important public health goals. Drinking plain water as a beverage instead of sugary drinks could help address obesity [1,2] as well as reducing the risk for Type II diabetes [3] and dental caries [4]. Drinking water can also help improve hydration status, potentially reducing the risk of wellbeing concerns such as headaches, stomachaches, and poorer cognitive function [5,6].

Ensuring adequate drinking water access is of particular concern for children’s health. Existing evidence suggests that over half of children and adolescents in the U.S. are inadequately hydrated at any given time, with children of color more likely to be inadequately hydrated [7]. Although several federal and state regulations require public schools in the United States to provide access to safe drinking water during the school day [8,9,10], evidence suggests that many schools do not provide adequate access to meet these regulations [9,11]. Additionally, lead contamination has proven to be a substantial problem in many school districts nationwide [12], particularly those with older school buildings and drinking water infrastructure.

School districts have thus struggled with how to provide drinking water to students while ensuring the water provided is safe to drink. Some school districts have shut off traditional tap water sources and begun providing bottled water coolers placed throughout the school building as an alternative [13,14]. However, little is known about how this type of system may impact students’ drinking water habits and perceptions. Little is also known about how perceptions of lead and other water quality concerns may influence student’s behavior and health and how school districts might successfully address water access and quality concerns.

The present study was conducted in partnership with colleagues from the Boston Public Schools (BPS), a large urban school district in the U.S. that has been working for decades to address the challenges of providing adequate water access with aging infrastructure. As part of district policy [15], BPS implements a rigorous annual testing of all water sources in each BPS building to assess lead levels in each tap water source and communicates those results to the community. BPS deactivates any individual unit with test results equal to or above 15 parts per billion (ppb) for lead, and when tests for specific units show lead concentrations greater than 1 ppb, BPS takes action to reduce lead concentration to the lowest possible level. As a result, some schools provide tap water access, with updated bottle filler/fountain drinking water units installed with wall-mounted filters, but most schools do not provide tap water for drinking and only provide bottled water from coolers.

We conducted a mixed-methods study using quantitative observations of drinking water access, observations of drinking water intake, surveys about students’ perceptions of water, as well as qualitative focus groups with students and school staff about water, to develop a comprehensive understanding of the impact of different systems for delivering water to students. Specifically, we aimed to: (1) describe water access and consumption in a small sample of schools, some with tap water access and some with only cooler water access; (2) explore students’ and staff members’ perspectives on the benefits and challenges of different water infrastructure systems; and (3) analyze quantitative and qualitative data concurrently to explore whether different delivery systems (coolers versus tap water) were related to consumption and student perceptions of water quality.

## 2. Methods

**Figure 1:**
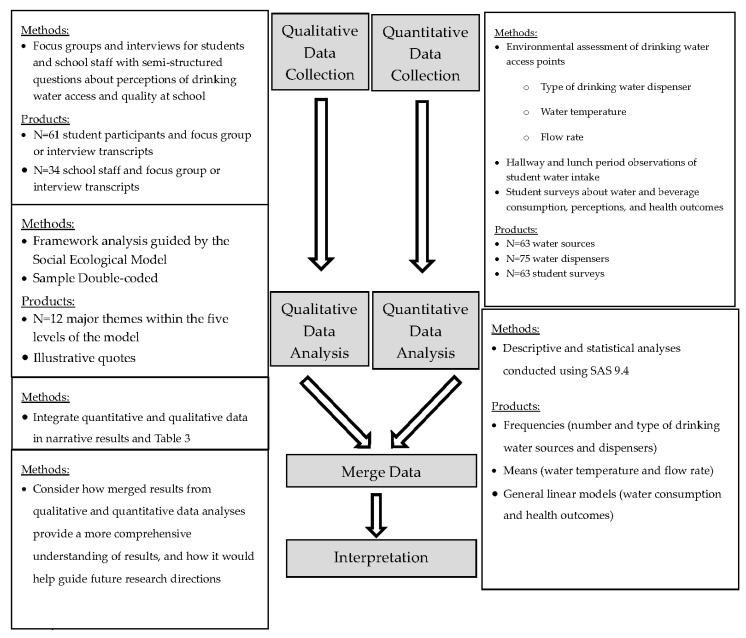
Convergent parallel mixed methods design of water access and students’ perceptions of water in 6 schools, 2018. Modified from Creswell and Plano Clark, 2011.

Study Design. To address our study aims, we utilized a concurrent mixed methods study design [16]. In this approach, quantitative and qualitative data are collected simultaneously, analyzed separately, then combined and compared in order to provide a richer description of the construct under study [16]. At six schools, we collected quantitative data on the quality and availability of drinking water access, students’ average water consumption, and students’ perceptions of water availability and quality via a brief survey. We collected qualitative data from both students and staff at the same schools via separate focus groups to better understand their perceptions of water quality and access and to explore how the physical environment and structure of the drinking water delivery system might impact students’ and staff members’ water perceptions, consumption habits, and wellbeing (Figure 1).

Sample. Our goal was to sample several schools within our partner district, ensuring that two different drinking water infrastructure systems prevalent in the district were represented across our sampled schools: (1) no tap water infrastructure available, with only bottled water coolers (i.e., coolers often seen in office settings with a base upon which a 5 gallon disposable carboy sits); and (2) tap water infrastructure available, including updated water fountain/bottle filler units. Our partners at the school district prepared a list of schools with these different infrastructure systems using their own administrative data. The research team then refined the list to be a group of schools that were roughly comparable on grade level and sociodemographic makeup. Our partners sent a letter to leaders of these schools introducing the study, and then research assistants followed up with direct phone calls and e-mails to invite schools to participate. After a school had agreed to participate, researchers scheduled a two-day period during which the quantitative observational data measuring school-level water access and average intake could be collected and scheduled two student and one staff focus groups. During the student focus groups, participants were also asked to complete surveys collecting quantitative data on self-reported water intake, perceptions of water access, and wellbeing. In instances when staff or students were not available for focus groups due to scheduling problems, one-on-one interviews were conducted. In total, we conducted nine focus groups plus three one-on-one interviews with students and four focus groups plus eight one-on-one interviews with school staff. To recruit students and staff for the focus groups and surveys, we distributed flyers about the study with each school secretary and/or a teacher or staff member involved in promoting wellness at the school. This study was approved by the Harvard T.H. Chan School of Public Health Institutional Review Board and the Boston Public Schools’ Office of Data and Accountability.

## 3. Measures

### 3.1. Quantitative Measures

Water access and quality. To assess how many functioning drinking water sources were in each school, researchers used a standardized instrument developed in previous studies [17]. Researchers walked through the school building with a school administrator and, at each drinking water source, recorded the location, type, temperature (using a digital thermometer, to the nearest tenth of a degree Fahrenheit), flow rate (the amount of time, in the nearest tenth of a second, it took to fill an 8 ounce Oxo measuring cup), functional status, and whether the source appeared clean to the researcher; if the researcher’s opinion was that it was not clean, s/he was asked to specify how so (e.g., mold, debris in basin). Researchers also noted whether cups were available.

Water intake. During lunch, researchers positioned themselves in the cafeteria so that it would be possible for one researcher to unobtrusively observe the cafeteria’s water source(s) and another to count the total number of students present in the cafeteria. Each student who drank school-provided drinking water was tallied, and each student in the cafeteria was counted in order to be able to estimate the proportion of students drinking water during each lunch period. When students used tap water sources such as water fountains or bottle fillers, researchers used stopwatches to time how long they had the fountain/bottle filler turned on and were either filling their cup/bottle or consuming the water directly from the source. Then, we calculated how much water was drawn for each student by multiplying this length of time by the flow rate of the fountain/bottle filler taken from the water access observation described above [17]. When students drank directly from a fountain rather than filling a cup or bottle, we further assumed that they were only able to consume 35% of the water flowing out of the fountain based on prior studies [17,18] and that they consumed 96% of water that was filled into cups or bottles. For non-tap water units, such as water coolers, research assistants weighed coolers at the beginning and end of each lunch period to quantify the weight of water taken, then divided this by the number of students observed taking water from that cooler to calculate the average student intake for each lunch period. These methods have been used previously [17].

To observe how much students consumed from school drinking water sources outside of the lunch area and outside of meal times, researchers chose one drinking water source elsewhere in the school to observe for a thirty minute period, using similar techniques to those described above (counting the number of students observed drinking water during that period and estimating the amounts of water consumed by weight for cooler or by flow rate for fountains and fillers). Each research assistant would then move at the end of that thirty-minute period to another water source to begin another thirty-minute observation period. We calculated the proportion of the student body observed drinking water per period in the hallways by dividing the number of students observed drinking by the school’s total enrollment.

Other beverage intake. After completing the count of total students in the cafeteria, researchers then conducted a scan of other beverages at student’s tables, recording, for as many students as possible, whether they had either no beverage at all, school-provided milk (flavored or plain), school-provided water, or a beverage from outside the school, including bottled water, 100% juice, sugar-sweetened beverages, or other beverages.

Individual student reports of water access and intake. A brief survey was distributed to students who participated in the focus groups to gather additional data on their perceived usual water intake (adapted from previously developed measures [19,20], perceptions of adequacy of water access in the school, and whether they had a headache, a stomachache, or difficulty concentrating in the last day. The goal of the survey was to assess whether students presented symptoms that have been linked with poorer hydration status in prior research [5,6] and were also of concern among health and wellness leaders in the school district where the study was conducted. Survey questions are included in Supplementary Materials.

School Demographics. Data on the sociodemographic makeup of the student body at each school was obtained from publicly available school administrative records.

### 3.2. Qualitative Measures

Student perceptions of water access and quality. Student focus groups and interviews were conducted using a semi-structured focus group guide, which included questions about students’ attitudes towards water, their perceptions of its taste, quality, and safety, their experiences with accessing water at school, and their ideas about water and health. The questions were developed in partnership with health and wellness leaders within the partner school district and also based on earlier studies of students’ perceptions of drinking water [21].

Staff perceptions. Staff focus groups and interviews were conducted with a separate semi-structured guide, including questions on what the school staff members saw as barriers to drinking water access at the school, as well as ideas for improvements. The questions were also developed in partnership with the partner school district health and wellness and facilities management leaders.

### 3.3. Analytic Approach

Quantitative Statistical Analysis. For each school, we calculated the average proportion of students consuming school-provided drinking water at each lunch period (number observed consuming divided by number observed in the cafeteria during a lunch period) stratified by water source type (bottled water coolers, traditional water fountains, and water station with fountain and bottle filler). We calculated the average amount of water consumed (oz) per student consuming water during the lunch period by water source and dispenser type. Similarly, for the hallway observations, we calculated the average proportion of the student body observed drinking water from the hallway water source per half hour observation period, as well as the average amounts of water consumed per student consuming water during each period. From the survey data, we classified students into low water consumers (drinking water during lunch a few times per month, once a month or less, or never) and high water consumers (drinking water during lunch every day, 3–4 days a week, or 1–2 days a week). Chi squared tests were used to determine significant differences between low versus high lunchtime water consumers and water access norms and health outcomes. All analyses were conducted in SAS v 9.4 (Cary, NC, USA, SAS Institute).

Qualitative Analysis. Focus groups and interviews were audio-recorded and transcribed verbatim. We conducted a framework analysis [22] grounded in the social-ecological model [23] to understand students’ and staff members’ perceptions of school drinking water in relation to the influence of factors within their schools, the school district, and surrounding community. First, two reviewers (JD and RL) analyzed one student and one staff focus group utilizing deductive coding approaches to categorize content within five domain areas of the social-ecological model (policy and environmental; community; organizational; interpersonal; individual) [23]. Next, inductive coding was used to develop constructs and sub-constructs within these five domain areas. A codebook was then developed and shared with other members of the research team to gather feedback and define agreed upon constructs. The same two reviewers proceeded to double-code one student and one staff focus group from each of the remaining schools, reconciling codes, and revised the codebook as appropriate. The remaining transcripts were coded by a single coder (JD). Analyses were conducted using NVivo qualitative data analysis software Version 11 and then organized according to the framework to summarize and condense into salient themes.

## 4. Results

Among the six schools, five served pre-kindergarten through eighth grade students, and one served sixth to twelfth graders with an average enrollment of 622 (SD ± 276.6) per school. On average, the student body at the schools was 57.0% Hispanic and 26.0% non-Hispanic Black; 66.0% were eligible to receive free or reduced-price meals. Four schools offered bottled water coolers only, one school had access to tap water only, and one school had access to both tap water and bottled water coolers. (Table 1)

Across the six schools, we observed 75 water dispensers in total; 46 coolers (61.3%), 17 fountains (22.7%), and 12 bottle fillers (16.0%). Six (8%) of water dispensers had no water at the time we observed them because they were either empty (*n* = 5) or broken (*n* = 1). The percentage of water sources with no stocked cups ranged dramatically by school (0% to 100% of coolers per school). Four (66.7%) of schools met the 75:1 student to water source ratio for Massachusetts state plumbing code, and four (66.7%) met a requirement of the National School Lunch Program to provide free drinking water access in meal services areas during lunch. (Table 1)

During two consecutive lunch observation days in the four schools where there was lunchtime water access, we observed 3751 students attending 37 lunch periods over 2 days of observation per school; in one school cafeteria, there was one water station and one water fountain, and in three school cafeterias, there were four coolers. Overall, the proportion of students who opted to drink water during lunch was low, ranging from 0% to 50% across the schools with bottled coolers and from 0% to 10% across schools with water fountain and water stations. Bottled water coolers and fountain components of tap water stations were more popular than traditional fountains (Table 2). During lunch, students who consumed water consumed, on average, 2.4 oz (SD ± 1.1) of water when drinking from coolers, 2.0 oz (SD ± 1.4) from fountains and when drinking from water stations with bottle fillers and fountains combined, 2.1 oz (SD ± 1.5) from the fountain and 26.5 oz (SD ± 25.3) from a filler, primarily because water was filled into bottles. During the lunch period, 186 (3.6%) of all students observed attending lunch in the cafeteria brought sugar-sweetened beverages from outside of the school to the lunch period, and 491 (13.1%) students consumed no beverage at all.

In observations of drinking water intake from hallway water sources outside of lunchtime (i.e., between or during classes), we conducted 12 half-hour observations in the hallways across the six schools. For bottled water coolers in school hallways, we observed about 4.1% of the student body consuming water on average during these observation periods. For traditional water fountains, we observed about only 0.4% of the student body, on average, consuming water. With updated bottle filler/fountain combination units, we observed 15.1% of the student body consuming water from the fountain components on average and 2.0% consuming from the bottle filler component.

**Figure 2:**
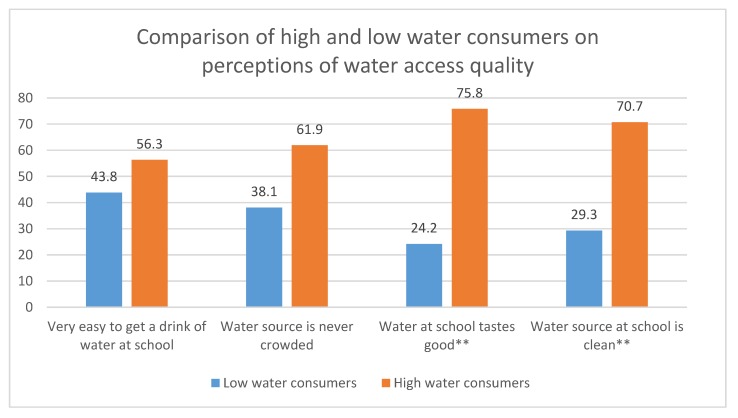

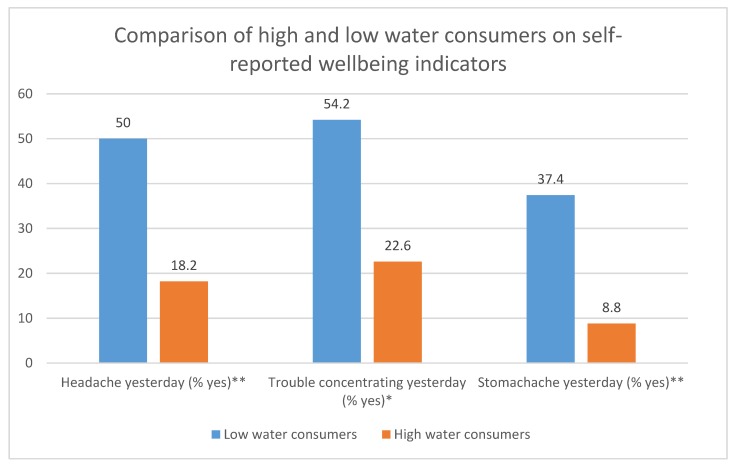
Comparison of perceived water access and perceived health survey responses by self-reported high and low water consumers across six urban schools, 2018 (*n* = 63 student respondents). * *p* < 0.05.** *p* < 0.01.

Sixty-three students completed the drinking water survey. While students who were high water consumers did not differ from those who reported consuming little water in their report of how easy it was to get a drink of water or how crowded their water sources were, taste and cleanliness appeared to be strongly linked with self-reported water consumption. High water consumers were significantly more likely to report that water consumed at school tasted good or very good compared to low water consumers (75.8% compared to 24.1%, *p* = 0.002) and that the water source at school was clean or very clean (70.7% compared to 29.3%, *p* = 0.003). Lower water consumption also was associated with worse self-reported wellbeing outcomes: students who were high water consumers were less likely to have headaches, difficulty concentrating, and stomachaches compared to low water consumers (*p* < 0.05 for all). (Figure 2)

### 4.1. Qualitative Results

Findings from the qualitative analysis are organized by theme within the five domain areas of the Social Ecological Model [23]. Similar themes often emerged among students and school staff, and at schools with tap water systems versus coolers. However, when discordant views emerged between groups, a detailed description of these differing perspectives is provided. Among the six schools, one had only student focus group participants and one had only staff. Of the 63 students who completed the survey, 61 participated in the focus groups. Thirty-four staff members participated in focus groups.

### 4.2. Policy and Environmental

#### School District Policy Implementation Challenges

Although the school district developed and adopted a comprehensive policy on water access in 2016 [15], which stipulated that all water sources used for drinking, food preparation, or medical services would be tested for lead annually, reports would be made available on the district’s website, updates to drinking water infrastructure would be undertaken over a ten year period, and students would learn about the benefits of drinking water in school, very few staff focus group participants knew of the district water policy, indicating a need for better communication.

Staff suggested that fidelity to district wellness initiatives, including reducing sugary drink consumption, varied considerably across schools. Some staff also expressed frustration at being expected to enforce policies limiting sugary drinks in school when water access and quality was inadequate. Similarly, some students believed that teachers and staff enforcing restrictions on sugary drinks were acting unfairly given their limited access to drinking water.

### 4.3. Community

#### 4.3.1. Neighborhood Influences

Both students and staff expressed that it is common for students to purchase sugary drinks at neighborhood stores and attempt to consume them during school time. Several staff members noted the challenge of having multiple fast food chains and retail stores in such close proximity to schools when trying to promote water to students.

#### 4.3.2. Relying on Outside Partnerships to Support Water Delivery

Staff at multiple schools with only coolers reported relying on youth or adult volunteers from community-based groups that supported school activities during the day or shared the space after school hours to restock the coolers. Additionally, staff described leveraging external partnerships to fund distribution of reusable water bottles.

### 4.4. Organizational

#### 4.4.1. Adequacy of Water Sources in Schools

Most students and staff indicated that the number of water sources available at their schools was not sufficient to meet student demand and did not provide adequate access. This deficiency was described as particularly noticeable in the cafeterias, where two schools did not have any water access. Even at schools with two cafeteria coolers, long lines and overcrowding were viewed as barriers to access for students. The transition following physical education classes or recess was another common example among students and staff highlighting the lack of water access. The heightened demand among students reportedly led to long lines at water sources between classes and insufficient time for students interested in accessing these sources. Some students cited the long lines and lack of time between classes as a reason they might not drink water during the school day.

#### 4.4.2. Stocking and Supplies Challenges

In schools with only coolers and no tap water sources, most students and staff reported frustration with how frequently they were empty. Coolers were sometimes described as needing to be refilled multiple times during a single school day, an onerous process that requires moving the over 40-pound, five-gallon commercial bottled water from a storage area to the coolers in need of restocking, and then lifting, flipping, and inserting it into the cooler units. Schools lacked any formal procedures for restocking coolers, so it was often left to students and staff to volunteer, sometimes during instructional class time. These were major reasons cited by students and staff in support of moving to a plumbed water delivery system.

Another stocking and supply issue reported by students and staff was the lack of disposable paper cups. While coolers and bottle fillers both support the use of reusable bottles, most students depend on disposable paper cups to drink water at school. Again, students and staff reported frustration with how frequently cups were inaccessible, which also added to time expended searching for water during class time. These cups, which hold 3 ounces of water, were also perceived to be too small.

#### 4.4.3. Safety and Cleanliness

Water sources, particularly coolers, perceived as dirty or unsanitary discouraged some students from drinking water at school. Some staff also suggested that coolers, which are prone to spills once the basin fills, create safety hazards. Students and staff supported their concerns about cooler cleanliness and safety with vivid accounts of cooler spigots being broken or clogged, basins filled with stagnant water and turned a pink hue, and visible particles floating inside the cooler jugs. Many students and staff also perceived water fountains to be unsanitary, mainly from having seen others put their mouth on the spout. Similarly, there were concerns about the cleanliness of cups given they were often left out in stacks on tables or nearby shelves, resulting in multiple people handling them before use.

School staff members were typically unaware of any existing protocols for the cleaning and maintenance of coolers and one teacher recounted a time when a cooler malfunctioned due to a rodent being electrocuted inside the cooler. While a replacement unit was provided in this instance, staff perceived attaining additional or replacement units on a regular basis to be challenging.

#### 4.4.4. Perceptions of Water Safety and Relationships with Water Infrastructure

While most students agreed that switching from coolers to a plumbed system with bottle fillers would improve water access, without filters, concerns about water quality would persist. Most staff perceived plumbed bottle filler/water fountain combination units as a superior alternative to water coolers or fountains both in terms of improving access and quality. However, a few staff discussed their own distrust of tap water and lingering concerns about lead contamination. Lastly, most students expressed a strong preference for cold water sources and staff confirmed that this often influenced which water sources students elected to drink from.

#### 4.4.5. Need for Further Education and Promotion

Staff indicated a need for additional school-based water education efforts related to health and safety, to both encourage drinking water and dispel lingering fears about potential lead contamination that could decrease consumption among students at schools transitioning to a plumbed water delivery system. While students and staff were largely supportive of efforts by schools to distribute reusable bottles, perceiving it as a useful strategy to increase access during class time, students tended to frequently lose track of them. Staff members were unsure how to effectively promote reusable bottle use given students’ tendency to lose them or forget to bring them to school and expressed concerns about keeping reusable bottles sanitary over prolonged periods of time.

### 4.5. Interpersonal

#### Staff Efforts to Promote Water Are Limited

Staff members described a variety of ways in which they encourage students to drink water, including verbal reminders, modeling behavior, promoting reusable bottle use, and making water accessible in the classroom using pitchers and water pumps. However, there was also near consensus among staff that students did not have sufficient access to drinking water and were not adequately hydrated during the school day. At the same time, many students indicated that teachers did not provide adequate opportunities to drink water during class time if it required leaving the classroom.

### 4.6. Individual

#### 4.6.1. Positive Views toward Drinking Water among Students

Students perceived drinking water, particularly in contrast to sugary drinks, to be a healthy behavior and associated a range of benefits, including adequate hydration and improved concentration with water consumption. Some staff expressed the view that students were well-informed regarding the health benefits of drinking water and increasingly aware of their water consumption habits. However, other staff suggested that many students lacked understanding about their hydration status and the importance of drinking water.

#### 4.6.2. Distrust of Tap Water

While some students did not hold any negative views toward tap water and considered it to be similar in quality to bottled water, many others held negative attitudes toward drinking tap water and perceived it to be dirty. This was a general view of tap water quality and not specific to the school setting. Students had a sophisticated understanding of the issues of lead-contaminated drinking water and leaching from aging pipes, describing them as a primary source contributing to these negative views. Accordingly, students often expressed a preference for bottled over tap water sources and stressed using filters when drinking from tap water sources to improve cleanliness. These students viewed drinking from filtered tap water sources more positively and similar in quality to bottled water.

### 4.7. Mixed Methods Integration

Table 3 presents the most salient themes from the qualitative analysis side-by-side with quantitative data and illustrative quotes to develop a comprehensive understanding of the complex issue of school water access. In integrating the qualitative and quantitative data, we found that several themes were reinforced across both data sources. The theme of not having enough water sources in schools, which came up several times in focus groups with staff and students, was reflected in our observational finding that 1/3 of the schools did not meet state or federal water provision regulations at the time of our visit and from our survey finding that 24% of students found it somewhat or very hard to get a drink of water at school. The theme of frequent emptiness and supply issues from the focus groups was echoed by our finding that 59% of water dispensers did not have cups at the time of our visit. Other themes appeared to have discordant findings between the quantitative and qualitative data. For example, while 95% of students reported on their surveys that they felt their teachers wanted them to drink water, and while teachers also repeatedly noted their wish for students to stay hydrated, in focus groups, students reported that teachers often prohibited them from drinking water.

## 5. Discussion

Our study, a comprehensive mixed-methods investigation across a small number of schools, suggests that schools have several serious challenges for providing adequate access to safe drinking water for students. Aging infrastructure, perceptions of inadequate availability of water sources, and perceptions of unsafe water quality appear to have contributed to very low student water intake, particularly in schools with traditional fountains. Although bottled water coolers have been used as a short-term solution when tap water infrastructure is found to have lead, our study suggests that this is not a tenable long-term solution because of the difficulty in consistently stocking cooler units in school settings and maintaining the units’ cleanliness. Our study participants even noted that the difficulties in keeping coolers stocked ended up interfering with students’ class time. At the same time, however, our quantitative observations suggest that students appeared to be more likely to drink from coolers than traditional tap water fountains or bottle filler/fountain units. While more modern water stations with bottle filler/fountain combination units were generally perceived positively in qualitative observations, their usage was not as high as that which was observed for coolers, suggesting that upgrades to drinking water infrastructure alone may not be sufficient without accompanying promotion, provision of adequate time to drink, and procedures to improve the appeal of the water through improved cleanliness. Similarly to prior qualitative and quantitative investigations of school staff and student perceptions of drinking water [21,24,25,26,27], we found deep distrust of tap water safety. Perceptions of tap water being unsafe to drink persist, given both the district’s local history in confronting lead-tainted water and recent national attention to lead contamination. Our results suggest that an intensive, strategic educational and promotion intervention strategy will likely be needed to build trust in tap water once safe tap water access is restored.

Our study is a unique contribution to the existing literature on school drinking water infrastructure, investigating how different solutions to aging water infrastructure challenges have played out in a school district. Several school districts have turned to providing bottled water coolers in the face of finding out that they have lead contamination [13,14]. Our study in a school district that has been implementing this system for decades suggests that this is not an effective long-term solution due to issues around empty bottles, inconsistent stocking, and cleanliness concerns, which all contribute to situations where drinking water access can be limited. Maintaining bottled water coolers as a school’s primary drinking water source requires substantially more labor—stocking, ordering, cleaning, checking on the units, upkeep—than relying on tap water [8]. While very short-term intervention studies have suggested that providing bottled water coolers can increase student consumption [28,29], these studies do not tell us about what the implications are of using these as the only school water source for several years. Additionally, while bottled water must meet similar standards for contaminants as tap water upon leaving the distributor, there are no regulations specifying that bottled water quality must be evaluated once it is in school settings that would correspond to regulations requiring ongoing tap water testing. Given the maintenance concerns that students and staff raised, it may not be a given that bottled water has fewer quality concerns than tap water.

Better school drinking water access matters. Improved access increases consumption [17,30,31,32] and can reduce obesity risk among students [33], possibly through displacing sugary drink consumption [2]. Improved water consumption can also maintain children’s hydration status, potentially contributing to better wellbeing; in the current study, several school staff expressed their concern that low water consumption was leading to headaches, attention problems, or general malaise among students, while student surveys suggested that infrequent consumption was statistically significantly associated with a higher likelihood of headaches, stomachaches, and difficulty concentrating. Yet, despite water being essential for optimizing children’s health and sfunctioning during the school day, recent research from different areas of the United States has found that students—who must spend a substantial amount of their waking hours inside a school building—do not have universal access to free, appealing drinking water [30]; in one study, nearly 50% of schools did not meet federal and state regulations for minimum provision of access [11]. Meanwhile, national evidence has shown that over half of all youth are inadequately hydrated on any given day, with higher risk for children of color [7]. Our results are consistent with previous studies showing that average water consumption in the school setting is very low—not much more than 1 ounce per student during lunch periods [29]. Taken together and in context with prior research, our findings suggest the urgent policy need to adequately fund water infrastructure improvements in school buildings across the nation, as well as the need to research and implement effective strategies for rebuilding trust in tap water and increasing students’ choice and consumption of water as a beverage.

Strengths of this study include its mixed-methods design, which allowed us to gather a nuanced, multi-faceted evaluation of key issues our participating schools were facing for drinking water access. Our study has several limitations, however. A trade-off with the deeper investigation was that the sample size was small, limiting our ability to make generalizable statements about the district as a whole. Although the observations were conducted at the schools in the same season (late spring), there was some day-to-day variation in temperature and precipitation that could have impacted children’s thirst that we were unable to account for in this analysis. We also were unable to measure individual student water intake over the course of the day, including precise measurement of water consumed from reusable bottles and carried throughout the day. Additionally, this study was only conducted in one urban location, and our findings may not be generalizable to others, though it is notable that our qualitative findings do echo studies conducted in locations from Washington, DC to San Francisco, CA [21,27].

## 6. Conclusions

For school districts across the country that are facing difficulty providing adequate drinking water access due to aging water infrastructure, our study suggests that substantial investment is needed in both improving plumbing infrastructure and in developing strategies to rebuild trust and promote consumption. Simply shutting off taps, boarding them up, and installing bottled water coolers without updating infrastructure is not likely to provide adequate, meaningful drinking water access in the long run, and may introduce a host of new implementation problems.

## Figures and Tables

**Table 1:** Sociodemographic and water access characteristics of six schools in a large urban district, 2018.

	Mean (SD) or *N* (%)
Grade levels served	
Pre-K through grade 8	5
Grades 6–12	1
Average enrollment	622 (276.6)
Average percent female	47.9 (2.3)
Average percent African American	26.0 (23.2)
Average percent Hispanic	57.0 (29.3)
Average percent White	6.3 (4.7)
Average percent Asian American	8.0 (16.6)
Average percent English language learner	37.9 (22.3)
Average percent students with disabilities	17.7 (8.6)
Average percent low income students	66.0 (11.7)
Water infrastructure type by school	
Bottled water coolers	4
Tap water access only	1
Tap water and coolers	1
Number of water sources observed ^1^	63
Bottled water coolers (%)	46 (73.0)
Water fountains (%)	4 (6.4)
Water stations (%)	13 (20.6)
Average number of water dispensers per school (±SD)	21.4 (24.4)
Number of water dispensers without water (%)	6 (8.0)
Number of water dispensers appearing dirty (%)	7 (9.6) ^2^
Number of water dispensers without cups (%)	44 (58.7)
Number of water dispensers with promotional signage (%)	3 (4.0)
Average temperature by water dispenser type	
Bottled water coolers (±SD)	54.5 (12.2)
Fountain (±SD)	67.2 (6.8)
Filler (±SD)	64.4 (2.3)
Number (%) of schools meeting 75:1 students to water source ratio for state plumbing code	4 (66.7%)
Number (%) of schools meeting HHFKA federal policy for free drinking water in cafeteria	4 (66.7%)

^1^ Water sources refer to a single source, for example, cooler, fountain or water station. Water dispenser refers to the separate water dispensers within a given water station, which includes a fountain and filler. ^2^ *N* = 2 missing data.

**Table 2:** Water consumption and likelihood of consuming, by water source type in six schools in a large urban district, 2018.

	Bottled Water Coolers	Traditional Water Fountains	Water Station with Fountain and Bottle Filler
*LUNCH*			
Number of schools with water sources in lunchroom ^1^	3	1	1
Number of lunch periods observations across all schools	32	5	5
Average number of students in cafeteria per lunch period observed	106.8 (68.2)	44.8 (30.9)	44.8 (30.9)
Average percent of students consuming water from source	14.1 (12.5)	5.1 (2.9)	4.5 (3.2)
Average amount of water (oz) consumed per student	(*N* = 370 observations) 2.4 (1.1)	(*N* = 9 observations): 2.0 (1.4)	Fountain (*N* = 9 observations): 2.1 (1.5) Filler (*N* = 3 observations): 26.5 (25.3)
*HALLWAY*			
Number of schools with water sources in hallways ^2^	4	1	2
Number of different locations observed across all schools	16	1	2
Number of total observation days across all schools	8	2	2
Number of students observed consuming water from dispensers from all schools	172	2	Fountain *N* = 127 Filler *N* = 17
Percentage of total enrollment observed consuming water from dispenser over 2 days of observations per school	Percent: 4.1 (5.4)	Percent: 0.4 (0.0)	Percent fountain: 15.1 (1.1) Percent filler: 2.0 (0.1)
Average amount of water consumed from dispenser while in hallway	3.2 (2.6)	1.93 (1.06)	Fountain: 1.75 (1.93) Filler: 20.5 (14.7)

^1^*N* = four schools with water sources in lunchroom; three schools had a cooler and one school had a water station and a fountain in the lunchroom. Two schools had no water sources available in the lunchroom. ^2^ *N* = six school water sources in hallways for observation; one school had a water station and a fountain hallway observations.

**Table 3:** Integration of quantitative observation and survey data about drinking water at school with qualitative themes and illustrative quotations from a mixed methods study of six urban schools, 2018.

Qualitative Theme	Descriptive Quantitative Data	Illustrative Quotes
Not enough water sources in schools	1/3 of schools did not meet 75:1 students to water source ratio for state plumbing code & federal policy for drinking water in cafeteria 1/3 of students reported that it was a little or very crowded at the water source they used most often	*“We could use one more [cooler] on each floor and one more in the gym and the pool deck, and possibly two more in the caf, but that’s the other problem… I don’t think those devices were meant to see the kind of wear that they’re getting inside of a building with 950 kids… it takes us forever to get a replacement” (Staff)*
Frequent emptiness, stocking, supply shortages	59% of water dispensers did not have cups	*“There’s two problems from the water. It’s either full and there’s no cups or there’s cups and it’s not full.” (Student)*
Need for improvement in school water delivery systems	24% of students reported that it was somewhat or very hard to get a drink of water at school	*“After they come back from lunch they’re always like, ‘oh my God, I need water.’ Like everyone needs water suddenly when they come back from lunch and I’m thinking: you just had lunch, there should be bubblers [sic water fountains] and cups in the lunch room.” (Teacher)*
Safety and cleanliness issues	10% of water dispensers appeared dirty in observational data 35% of students reported that the water dispenser they use most often was not consistently clean	*“Yeah, I mean it’s [the water cooler] under contract and the only reason I know that is because, grossly, a mouse fried in one of them.” (Teacher)* *“I hate that other people put their mouths on the faucet so other people can get some water and get their germs.” (Student)*
Staff efforts to promote water are limited	95% of students reported they think their teachers want them to drink water	*“If I ask my teacher to get water and we’re in the middle of a class, she’s not going to let me until like after the class. Or if I’m on my way to lunch or something.” (Student)*
Positive views towards drinking water among students as well as a distrust of tap water	54% of students reported the water in the dispenser they use most often tastes good	*“I find that water is refreshing.” (Student)* *“Water is good for you cause like helps you grow and it set your mind straight.” (Student)* *“The pipes can probably be dirty and it’s probably been there for like long ages” (Student)*

## Supplementary Materials

The following are available online at https://www.mdpi.com/1660-4601/17/1/62/s1.
