# Supplementary material for: Providing Students with Adequate School Drinking Water Access in an Era of Aging Infrastructure: A Mixed Methods Investigation
Source: Int J Environ Res Public Health. 2019 Dec 20;17(1):62. doi: 10.3390/ijerph17010062 (PMC6981468; doi:10.3390/ijerph17010062)
Supplement: Supplementary file 1 [file ijerph-17-00062-s001.pdf]

Boston Public Schools: Survey about Drinking Water **Participant ID:_____________**

Instructions. Read each question below. Circle the response below each question that best matches your own answer. There are no right or wrong answers to these questions – we want to know what YOU think.

1. **During lunch, how <u>often</u> do you usually drink water? Circle one.**

Every day	3-4 days a week	1-2 days a week	A few times a month	Once a month or less	Never

2. **During lunch, how <u>much</u> water do you usually drink?**

A whole bottle	About a half a bottle	A whole plastic or paper cup	A half a plastic or paper cup	A few sips	I don't drink water during lunch

3. **During recess, gym, or PE, how <u>often</u> do you usually drink water? Circle one.**

Every day	3-4 days a week	1-2 days a week	A few times a month	Once a month or less	Never

4. **During recess, gym, or PE, how <u>much</u> water do you usually drink?**

A whole bottle	About a half a bottle	A whole plastic or paper cup	A half a plastic or paper cup	A few sips	I don't drink water during recess, gym or PE

5. **Between classes at school, about how <u>often</u> do you usually drink water?**

Every day	3-4 days a week	1-2 days a week	A few times a month	Once a month or less	Never

6. **Between classes at school, about how <u>much</u> water do you usually drink?**

A whole bottle	About a half a bottle	A whole plastic or paper cup	A half a plastic or paper cup	A few sips	I don't drink water between classes

7. **During lunch, how <u>often</u> do you usually drink a sugary drink? Sugary drinks include soda, lemonade or fruit punch, sports drinks (like Gatorade), energy drinks (like Monster or Red Bull), or sweetened iced tea or coffee (like a Frappuccino).**

Every day	3-4 days a week	1-2 days a week	A few times a month	Once a month or less	Never

8. **During lunch, how <u>much</u> of a sugary drink do you usually drink?**

A whole bottle or can	About a half a bottle or can	A few sips	I don't drink sugary drinks during lunch

9. **Between classes at school, about how <u>often</u> do you usually drink a sugary drink?**

Every day	3-4 days a week	1-2 days a week	A few times a month	Once a month or less	Never

10. Between classes at school, about how <u>much</u> of a sugary drink do you usually drink?

A whole bottle or can | About a half a bottle or can | A few sips | I don't drink sugary drinks between classes

11. How easy is it to get a drink of water at your school when you're thirsty? Circle one:

Very easy | Somewhat easy | Somewhat hard | Very hard

12. How crowded is it near the water fountain or cooler you use most often at school? Circle one:

Very crowded – I always have to wait in line | A little crowded – I sometimes have to wait in line | Not at all crowded – I never have to wait in line

13. Think about the water fountain or cooler you use most often at school. How does the water from this fountain or cooler taste?

Very good | Good | OK | Bad | Very bad

14. Think about the water fountain or cooler you use most often at school again. How clean is it?

Very clean | Clean | Somewhat clean | Dirty | Very dirty

15. Do you think your friends like to drink water at school?

Yes | No | Don't know

16. Do you think your family wants you to drink water at school?

Yes | No | Don't know

17. Do you think your teachers want you to drink water at school?

Yes | No | Don't know

18. Think about yesterday. Did you have a headache at any point during the day?

Yes | No | Don't know

19. Think about yesterday. Did you find it hard to concentrate at any point during the day?

Yes | No | Don't know

20. Think about yesterday. Did you feel very thirsty at any point during the day?

Yes | No | Don't know

21. Think about yesterday. Did your stomach hurt at any point during the day?

Yes | No | Don't know
